# *In vitro* antioxidant and anticancer effects of solvent fractions from *prunella vulgaris* var. *lilacina*

**DOI:** 10.1186/1472-6882-13-310

**Published:** 2013-11-09

**Authors:** Yu-Jin Hwang, Eun-Ju Lee, Haeng-Ran Kim, Kyung-A Hwang

**Affiliations:** 1 Department of Agrofood Resources, National Academy of Agricultural Science, RDA, Suwon, Gyeonggi-do 441-853, Republic of Korea; 2 Department of Biotechnology & Bioengineering, Sungkyunkwan University, Suwon, Gyeonggi-do 440-746, Republic of Korea

**Keywords:** *Prunella vulgaris* var. *lilacina*, Antioxidative activity, Anticancer activity

## Abstract

**Background:**

Recently, considerable attention has been focused on exploring the potential antioxidant properties of plant extracts or isolated products of plant origin. *Prunella vulgaris* var. *lilacina* is widely distributed in Korea, Japan, China, and Europe, and it continues to be used to treat inflammation, eye pain, headache, and dizziness. However, reports on the antioxidant activities of *P. vulgaris* var. *lilacina* are limited, particularly concerning the relationship between its phenolic content and antioxidant capacity. In this study, we investigated the antioxidant and anticancer activities of an ethanol extract from *P. vulgaris* var. *lilacina* and its fractions.

**Methods:**

Dried powder of *P. vulgaris* var. *lilacina* was extracted with ethanol, and the extract was fractionated to produce the hexane fraction, butanol fraction, chloroform fraction and residual water fraction. The phenolic content was assayed using the Folin-Ciocalteu colorimetric method. Subsequently, the antioxidant activities of the ethanol extract and its fractions were analyzed employing various antioxidant assay methods including DPPH, FRAP, ABTS, SOD activity and production of reactive oxygen species. Additionally, the extract and fractions were assayed for their ability to exert cytotoxic activities on various cancer cells using the MTT assay. We also investigated the expression of genes associated with apoptotic cell death by RT-PCR.

**Results:**

The total phenolic contents of the ethanol extract and water fraction of *P. vulgaris* var. *lilacina* were 303.66 and 322.80 mg GAE/g dry weight (or fractions), respectively. The results showed that the ethanol extract and the water fraction of *P. vulgaris* var. *lilacina* had higher antioxidant content than other solvent fractions, similar to their total phenolic content. Anticancer activity was also tested using the HepG2, HT29, A549, MKN45 and HeLa cancer cell lines. The results clearly demonstrated that the *P. vulgaris* var. *lilacina* ethanol extract induced significant cytotoxic effects on the various cancer cell lines, and these effects were stronger than those induced by the *P. vulgaris* var. *lilacina* solvent fractions. We also investigated the expression of genes associated with apoptotic cell death. We confirmed that the *P. vulgaris* var. *lilacina* ethanol extract and water fraction significantly increased the expression of p53, Bax and Fas.

**Conclusions:**

These results suggest that the ethanol extract from *P. vulgaris* var. *lilacina* and its fractions could be applied as natural sources of antioxidants and anticancer activities in food and in the pharmaceutical industry.

## Background

Oxidative stress is caused by reactive oxygen species (ROS), which are associated with many pathological disorders such as atherosclerosis, diabetes, ageing and cancer [1-3]. In order to protect human beings against oxidative damage, synthetic antioxidants such as BHA and BHT were created due to demand [4]. However, there has been concern regarding the toxicity and carcinogenic effects of synthetic antioxidants [5,6]. Thus, it is important to identify new sources of safe and inexpensive antioxidants of natural origin. Natural antioxidants, especially plant phenolics, flavonoids, tannins and anthocyanidins, are safe and are also bioactive [7]. Therefore, in recent years, considerable attention has been focused on exploring the potential antioxidant properties of plant extracts or isolated products of plant origin [8].

*Prunella vulgaris* var. *lilacina* is widely distributed in Korea, Japan, China, and Europe, and it continues to be used to treat inflammation, eye pain, headache, and dizziness [9,10]. It is rich in active compounds known to significantly affect human health, such as triterpenoid, rosmarinic acid, hyperoside, ursolic acid, and flavonoids [11-16]. Furthermore, *P. vulgaris* var. *lilacina* has been shown to have anti-allergic, anti-inflammatory, anti-oxidative, anti-microbial, and anti-viral effects [17-19]. However, reports on the antioxidant activities of *P. vulgaris* var. *lilacina* are limited, particularly concerning the relationship between its phenolic content and antioxidant capacity. Therefore, the aims of this study were to identify new sources of antioxidants from *P. vulgaris* var. *lilacina* extract. Additionally, the effects of the extraction solvent (70% ethanol, hexane, butanol, chloroform, or water) on the total phenolic content and antioxidant activities of *P. vulgaris* var. *lilacina* were investigated.

## Methods

### Reagents

The reagents 1,1-diphenyl-1-picrylhydrazyl (DPPH), 2,2′-azinibis 3-ethyl benzothiazoline-6-sulfonic acid (ABTS), α-tocopherol, 2,4,6-tris(2-pyridyl)-s-triazine (TPTZ), iron(III) chloride hexahydrate, gallic acid, Folin and Ciocalteu’s phenol reagent, 3-(4,5-dimethylthiazol-2-yl)-2,5-diphenyltetrazolium bromide (MTT) and lipopolysaccharides (LPS) were purchased from Sigma-Aldrich (St. Louis, MO, USA). Iron(II) sulfate heptahydrate and acetic acid were purchased from Junsei (Tokyo, Japan). A superoxide dismutase-WST kit was purchased from Dojindo (Kumamoto, Japan). Dulbecco’s Modified Eagle’s Medium (DMEM), RPMI 1640 medium, fetal bovine serum (FBS), and penicillin–streptomycin were obtained from Invitrogen (Carlsbad, CA, USA).

### Sample preparation and extraction

Whole plants of *P. vulgaris* var. *lilacina* were purchased from the Plant Extract Bank (#007-017, Dae-Jeon, Korea). Dried *P. vulgaris* var. *lilacina* was milled into powder of 80-mesh particle size and stored at -70°C. The dried *P. vulgaris* var. *lilacina* was extracted three times with 70% ethanol. The 70% ethanol extract powder (10 g) was suspended in 500 mL of distilled water and extracted with 500 mL of the following solvents in a stepwise manner: hexane, chloroform, and butanol. Each fraction was filtered through Whatman filter paper No. 2 (Advantec, Tokyo, Japan). Subsequently, the filtrates were combined and evaporated under a vacuum and then lyophilized with a freeze dryer (Ilshine Lab, Suwon, Korea) at -70°C under reduced pressure (< 20 Pa). The dry residue was stored at -20°C. For further analysis, we reconstituted the dry extract and fractions with DMSO.

### Total phenolic content

The total phenolic contents of *P. vulgaris* var. *lilacina* extract and its fractions were determined using the Folin-Ciocalteu method [20]. The extract and each fraction were oxidized with Folin–Ciocalteu’s reagents, and then, the reaction was neutralized with 10% sodium carbonate. After incubation at room temperature for 1 h, the absorbance of the reaction mixture was measured at 725 nm using a microplate reader (Molecular Devices, Sunnyvale, CA, USA). Quantification was performed based on a standard curve with gallic acid. Results were expressed as milligrams gallic acid equivalent (GAE) per gram of dry weight of extract (or fractions).

### DPPH radical scavenging activity

Analysis of DPPH radical-scavenging activity was carried out according to the Blois method [21]. 0.3 mM DPPH was added to each sample. After incubation for 30 min in the dark at room temperature, the absorbance was measured at 518 nm using a microplate reader. α-Tocopherol was used as a positive control. Percent reduction of the DPPH radical was calculated in the following way: inhibition concentration (%) = 100 - (A_sample_/A_control_) × 100,

where A_control_ is the absorbance of the control reaction (containing all reagents except the test sample), and A_sample_ is the absorbance of the test sample. Tests were carried out in triplicate. For the final results, RC_50_ values (the concentrations required for 50% reduction of DPPH by 30 min after starting the reaction) were calculated from the absorbance diminished by 50%. The experiment was performed in triplicate.

### Ferric-reducing antioxidant power (FRAP) activity

FRAP activity was determined using manual assay methods [22]. The working fluid was freshly prepared by mixing acetate buffer (300 mM, pH 3.6) with TPTZ in HCl and iron (III) chloride hexahydrate. Each sample solution or α-tocopherol was added to 3 mL of working fluid, and the mixture was left for 4 min at room temperature. The absorbance was measured at 593 nm. The results were expressed as iron (II) sulfate heptahydrate (FeSO_4_) equivalents.

### ABTS radical cation scavenging activity

The ABTS assay was based on the ability of different fractions to scavenge the ABTS radical cation in comparison to a standard (α-tocopherol) [23]. The radical cation was prepared by mixing 7 mM ABTS with 2.45 mM potassium persulfate (1:1 v/v) and leaving the mixture for 24 h until the reaction was completed and the absorbance was stable. The ABTS radical solution was diluted with PBS to an absorbance of 0.7 ± 0.02 at 732 nm. The photometric assay was conducted with 180 μL of ABTS radical solution and 20 μL of samples; measurements were taken at 732 nm after 1 min. The antioxidative activity of the tested samples was calculated by determining the decrease in absorbance. The free radical scavenging capacity was expressed by RC_50_.

### Superoxide dismutase (SOD) activity

Superoxide dismutase activity was determined using the highly water-soluble tetrazolium salt WST-1, which produces a water-soluble formazan dye upon reduction with a superoxide anion. SOD activity was determined using an SOD assay kit (Dojindo, Kumamoto, Japan) in accordance with the manufacturer’s instructions. Briefly, WST working solution was made by diluting 1 mL of WST solution into 19 ml of buffer solution. Enzyme working solution was made after the enzyme solution tube was centrifuged for 5 sec. Fifteen microliters of enzyme solution were diluted with 2.5 mL of dilution buffer. SOD activity was expressed as the percentage of inhibition rate.

### Cells and culture

The mouse macrophage cell line RAW264.7, human liver cancer cell line HepG2, human colon cancer cell line HT29, human lung cancer cell line A549, human stomach cancer cell line MKN-45 and human cervical cancer cell line HeLa were purchased from the Korean Cell Line Bank (Seoul, Korea). The cell lines were grown in RPMI 1640 medium or DMEM with 10% FBS and 1% penicillin-streptomycin and incubated at 37°C in 5% CO_2_.

### Cell cytotoxicity assay

Exponentially growing cells were collected and plated at 5 × 10^3^ - 1 × 10^4^ cells/well. *P. vulgaris* var. *lilacina* ethanol extract and its solvent fractions in DMSO were diluted in PBS to obtain final concentrations of 10, 50 and 100 μg/mL. Cells were treated with samples for 24 h, and MTT solution was added. After 4 h, the media was removed, and DMSO was added to each well. The resulting absorbance was measured at 540 nm [24].

### Intracellular reactive oxygen species (ROS) scavenging activity

For microscopic detection of ROS formation, RAW264.7 cells were grown to 80% confluence in six-well plates and treated with samples for 24 h. After incubation, cells were incubated with dichlorofluorescein diacetate (DCF-DA) (25 μM) for 30 min at 37°C in the dark. After several washings with PBS, cells were observed with a fluorescence microscope (Carl ZEISS, Oberkochen, Germany).

### Real-time reverse transcription polymerase chain reaction analysis (RT-PCR)

To determine the expression levels of p53, Bax, Bcl-2 and Fas, RT-PCR was performed using a Qiagen Rotor-Gene Q real-time thermal cycler (Valencia, CA, USA) in accordance with the manufacturer’s instructions. The cells were treated with *P. vulgaris* var. *lilacina* extracts and cultured for 24 h. Thereafter, cDNA was synthesized from the total RNA isolated from cells. The PCR reaction was performed using 2× SYBR Green mix (Qiagen, Valencia, CA, USA). All results were normalized to glyceraldehyde 3-phosphate dehydrogenase (GAPDH) expression. The following primer sequences were used for the real-time RT-PCR: GAPDH, 5′-CGG AGT CAA CGG ATT TGG TCG TAT-3′ (forward), 5′-AGC CTT CTC CAT GGT GGT GAA GAC-3′ (reverse); p53, 5′-GCT CTG ACT GTA CCA CCA TCC-3′ (forward), 5′-CTC TCG GAA CAT CTC GAA GCG-3′ (reverse); Bax, 5′-ATG GAC GGG TCC GGG GAG-3′ (forward), 5′-TCA GCC CAT CTT CTT CCA-3′ (reverse); Bcl-2, 5′-CAG CTG CAC CTG ACG-3′ (forward), 5′-ATG CAC CTA CCC AGC-3′ (reverse); Fas, 5′- TCT AAC TTG GGG TGG CTT TGT CTT C -3′ (forward), 5′- GTG TCA TAC GCT TTC TTT CCA T-3′ (reverse).

### Gas chromatography-mass spectrum analysis (GC-MS)

GC-MS analysis was carried out using an Agilent 6890 gas chromatograph equipped with a DB-5 ms capillary column (60 m × 0.25 mm; coating thickness 1.4 μm) and an Agilent 5975 MSD detector (Loveland, CO, USA). Analytical conditions were as follows: injector and transfer line temperatures of 250°C; oven temperature was programmed from 50°C to 150°C at 10°C/min, from 150°C to 200°C at 7°C/min, and from 200°C to 250°C at 5°C/min; carrier gas helium at 1 mL/min; and split ratio 1:10. Identification of the constituents was based on comparison of the retention times with those of authentic samples.

### Statistical analysis

Statistical analysis was performed with SPSS (version 17.0; SPSS Inc., Chicago, IL, USA). Descriptive statistics were used to calculate the mean and standard error of the mean (SEM). One-way analysis of variance was performed, and when the significance (p < 0.05) was determined, the differences of the mean values were identified using Duncan’s multiple range tests.

## Results and discussion

### Extraction yield

The yield of the extract and each fraction obtained from dry plant material was measured (Additional file 1: Table S1). The highest solid residue yields were obtained using butanol as the extraction solvent.

### Total phenolic content

The total phenolic content of the *P. vulgaris* var. *lilacina* extract and its fractions were determined through a linear gallic acid standard curve and expressed as mg GAE/g dry weight of extract (or fractions). As shown Table 1, the total phenolic content of all fractions from *P. vulgaris* var. *lilacina* varied from 109.31 to 322.80 mg GAE/g. The highest total phenolic content was detected in the water fraction (322.80 ± 15.12 mg GAE/g), whereas the lowest content was found in the hexane fraction (109.31 ± 4.08 mg GAE/g). Phenolic compounds are reported to be associated with antioxidant activity, anticancer effects, and other biological functions and may prevent the development of aging and disease [25]. These results suggest that *P. vulgaris* var. *lilacina* extracts might have high antioxidant and anticancer activities.

**Table 1:** **Total phenolic contents of various solvent fractions obtained from the ethanol extract of *Prunella vulgaris *var**. ***lilacina***

**Solvent**	**Total polyphenoles**^**1)**^**(mg GAE/g)**
70% ethanol	303.66 ± 3.61^a^
Hexane	109.31 ± 4.08^c^
Butanol	242.03 ± 6.16^b^
Chloroform	124.45 ± 0.24^c^
Water	322.80 ± 15.12^a^

^1)^Total phenolic content expressed in mg of gallic acid equivalent (*GAE*) per gram of dry weight of extract (or fractions).

Values are mean ± *SEM*. Values with different superscripts within same column are significantly different (p < 0.05).

### Antioxidant capacities of *prunella vulgaris* var. *Lilacina*

Results of the radical scavenging capacities determined by DPPH, FRAP, ABTS and SOD assays are shown in Table 2. In the DPPH assay, the DPPH radical scavenging activity of all fractions from *P. vulgaris* var. *lilacina* extract increased as shown in Table 2; the RC_50_ values of radical scavenging activity for DPPH were found to be 73.05 ± 10.32, 1402.96 ± 194.46, 83.52 ± 7.01, 521.58 ± 8.10, 64.26 ± 2.22, and 12.82 ± 1.33 μg/mL for ethanol extract, hexane, butanol, chloroform, water fractions and α-tocopherol, respectively. The water fraction showed the highest DPPH radical-scavenging activity. The DPPH scavenging activity of all fractions showed a similar trend to the content of total phenolic compounds. The FRAP assay measures total antioxidant activity based on the reduction of the ferric tripyridyltriazine (Fe^3+^-TPTZ) complex to the ferrous form. The ferric complex reducing abilities of different fractions were similar to the results obtained for the radical scavenging assay; water fraction exhibited very strong ferric ion reducing activity, and the five fractions in descending order of strength of ferric ion reducing activity were water fraction > ethanol extract > butanol > chloroform > hexane fraction. In terms of the ABTS assay, the water fraction demonstrated the highest scavenging activity, followed by the ethanol extract, butanol fraction, chloroform fraction and hexane fraction, and these trends was similar to those of the DPPH and FRAP assays. For the results of the SOD activity assay, most fractions with the exception of hexane exhibited very high SOD activity similar to α-tocopherol (99.64 ± 2.45%). However, there was no significant different among the fractions.

**Table 2:** **Total antioxidant capacities of various solvent fractions of the ethanol extract of *Prunella vulgaris *var**. ***lilacina***

**Solvent**	**DPPH RH**_**50**_^**1)**^_**(ug/** **ml)**_	**FRAP value**^**2)**^_**(m M/** **g)**_	**ABTS RC**_**50**_^**3)**^_**(ug/** **ml)**_	**SOD activity**^**4)**^_**(%)**_
70% ethanol	73.05 ± 10.3^c^	1.07 ± 0.08^ab^	60.08 ± 3.19	87.74 ± 3.42
Hexane	1402.96 ± 194^a^	0.48 ± 0.09^c^	1469.12 ± 10.1^a^	23.80 ± 0.53
Butanol	83.52 ± 7.01^c^	0.95 ± 0.09^bc^	61.13 ± 4.23^c^	92.54 ± 0.32
Chloroform	521.58 ± 8.10^b^	0.65 ± 0.02^bc^	153.04 ± 0.58^b^	72.75 ± 3.91
Water	64.26 ± 2.22^c^	1.24 ± 0.12^a^	53.78 ± 2.60^c^	87.51 ± 3.20
a-tocoperol	12.82 ± 1.33	15.0 ± 26	7.54 ± 0.13	99.64 ± 2.45

^1)^Concentration of test sample required to produce 50% inhibition of the *DPPH* radical.

^2)^Expressed as mmol of Fe^2+^ equivalents per gram of dry plant weight.

^3)^Concentration of test sample required to produce 50% inhibition of *ABTS* radical.

^4)^Expressed as the superoxide inhibition rate of *Prunella vulgaris* var. *lilacina* ethanol extract and its fractions.

Values are mean ± *SEM*. Values with different superscripts within same column are significantly different (p < 0.05).

DPPH, FRAP, ABTS and SOD assays are widely used to determine the antioxidant capacity of plant extracts due to their simplicity, stability, and reproducibility [26]. In this study, the DPPH, FRAP, ABTS and SOD assays provided comparable results for the antioxidant capacity measured in *P. vulgaris* var. *lilacina* extract and its fractions. The *P. vulgaris* var. *lilacina* extract and its fractions exhibited strong antioxidant activities against various oxidative systems *in vitro*. The strong antioxidant activity of a plant extract is correlated with a high content of total phenols [27,28]. In our research, we observed that the *P. vulgaris* var. *lilacina* extract and its fractions that contained higher phenol content exerted stronger radical scavenging effects (Table 3). The correlations between the antioxidant assays, such as DPPH, FRAP, ABTS and SOD activity and phenolic content, were highly positive (0.759 < │r│ < 0.993, p < 0.01), indicating that the four assays provided comparable values when they were used to estimate the antioxidant capacity of *P. vulgaris* var. *lilacina* extract. Many studies have shown a good positive linear correlation between antioxidant capacity and the total phenolic content of spices, medicinal herbs, and other dietary plants. Moreover, these results have also suggested that phenolic compounds are responsible for their antioxidant capacity [29-31].

**Table 3:** **Correlation coefficients between the antioxidant capacity and phenolic content of various solvent fractions of the ethanol extract of *Prunella vulgaris *var**. ***lilacina***

**Factor**^**1)**^	**TPC**	**DPPH**	**FRAP**	**ABTS**	**SOD**
TPC	1.000	-0.834	0.982**	-0.673	0.764
DPPH		1.000	-0.886	0.962**	-0.993**
FRAP			1.000	-0.759	0.828
ABTS				1.000	-0.980**
SOD					1.000

^1)^*TPC*: total phenolic content, *DPPH*: *DPPH* radical scavenging activity, *FRAP*: ferric reducing antioxidant powers, *ABTS*: *ABTS* radical scavenging activity, *SOD*: Superoxide dismutase activity. ^*^p<0.05, ^**^p<0.01.

### Intracellular ROS scavenging activity

**Figure 1:**
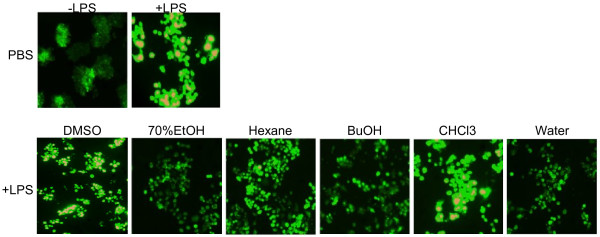
**Reactive oxygen species scavenging activities of *Prunella vulgaris *var. *lilacina*.** Cells were treated with solvent fractions of *Prunella vulgaris* var. *lilacina* at 10 μg/mL. After treatment for 24 h, ROS was stained with a DCF-DA for 30 min, and the generation of ROS was analyzed with fluorescence microscopy.

To investigate the intracellular levels of ROS, the cell-permeable probe DCF-DA was utilized. Non-fluorescent DCF-DA, hydrolyzed to DCFH inside the cells, yields highly fluorescent DCF-DA in the presence of intracellular hydrogen peroxide and related peroxides [32]. We examined whether *P. vulgaris* var. *lilacina* extract and its fractions inhibited LPS-induced ROS generation. As shown in Figure 1, LPS treatment significantly increased ROS formation in RAW264.7 cells as determined by DCF fluorescence. However, treatment with *P. vulgaris* var. *lilacina* extract and its fractions blocked LPS-induced ROS generation similar to the results obtained for the antioxidant assays.

### Cell cytotoxicity activity

**Figure 2:**
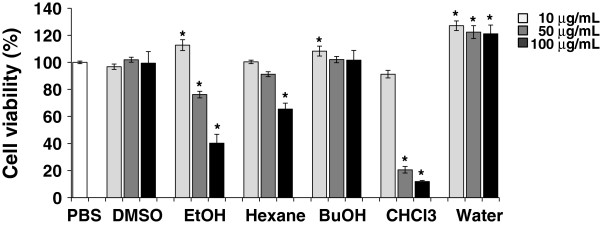
**Effects of *Prunella vulgaris *var. *lilacina *on RAW264.7 cells as determined by the MTT assay.** Cells were treated with solvent fractions of *Prunella vulgaris* var. *lilacina* at different concentrations (10, 50 and 100 μg/mL). After treatment for 24 h, cell viability was measured with the MTT assay. Values are the mean ± SEM; different marks within treatments indicate significant differences at *p < 0.05 compared to the PBS group.

In order to evaluate the cytotoxic effects of all samples, we performed a preliminary cytotoxicity study with RAW264.7 cells exposed to various sample concentrations (10, 50, or 100 μg/mL). The *P. vulgaris* var. *lilacina* ethanol extract (at 50 and 100 μg/mL), hexane fraction (at 100 μg/mL) and chloroform fraction (at 50 and 100 μg/mL) inhibited cell proliferation, but did not at a concentration of 10 μg/mL (Figure 2). Conversely, the groups treated with 10 μg/mL of ethanol extract or butanol fraction or treated with 10, 50 and 100 μg/mL of the water fraction showed a proliferative effect of over 10%. Macrophages are specialized phagocytic cells that attack foreign substances and cancer cells through destruction and ingestion. They also stimulate lymphocytes and other immune cells to respond to pathogens [33]. These results suggest that the ethanol extract, butanol and water fractions of *P. vulgaris* var. *lilacina* can be used in the treatment of cancer. Based on this result, we determined the appropriate concentration to be 10 μg/mL.

**Figure 3:**
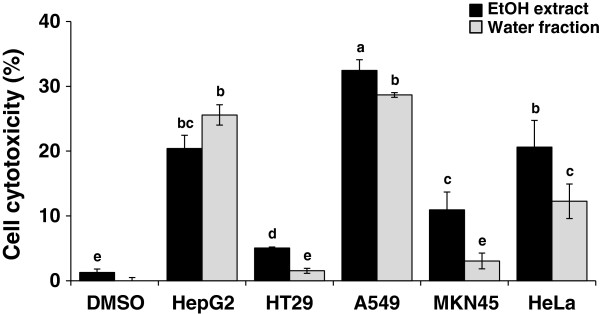
**The cell cytotoxicity of the ethanol extract and water fraction from *Prunella vulgaris *var. *lilacina *against various cancer cell lines.** Cells were treated with the ethanol extract and water fraction of *P. vulgaris* var. *lilacina* at 10 μg/ml. After treatment for 24 h, cell viability was measured with the MTT assay. Values are the means of three determinations ± SEM. The different letters indicate a significant difference of *p* < 0.05.

Involvement of free radical-mediated cell damage in many different diseases, particularly cancer, led us to evaluate the cytotoxic activities of the ethanol extract and the water fraction of *P. vulgaris* var. *lilacina* against five human cancer cell lines (liver, HepG2; colon, HT29; lung, A549; stomach, MKN-45; and cervical, HeLa) (Figure 3). The ethanol extract and the water fraction of *P. vulgaris* var. *lilacina* were most effective on A549 out of all the cancer cell lines; their values were 32.4 and 28.7% at 10 μg/mL, respectively.

**Figure 4:**
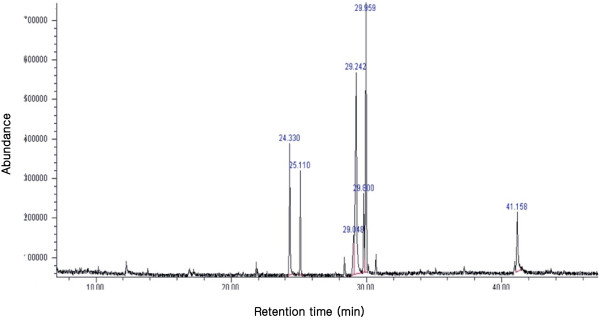
**Gas chromatogram of the ethanol extract of *Prunella vulgaris *** **var. *lilacina*.**

**Figure 5:**
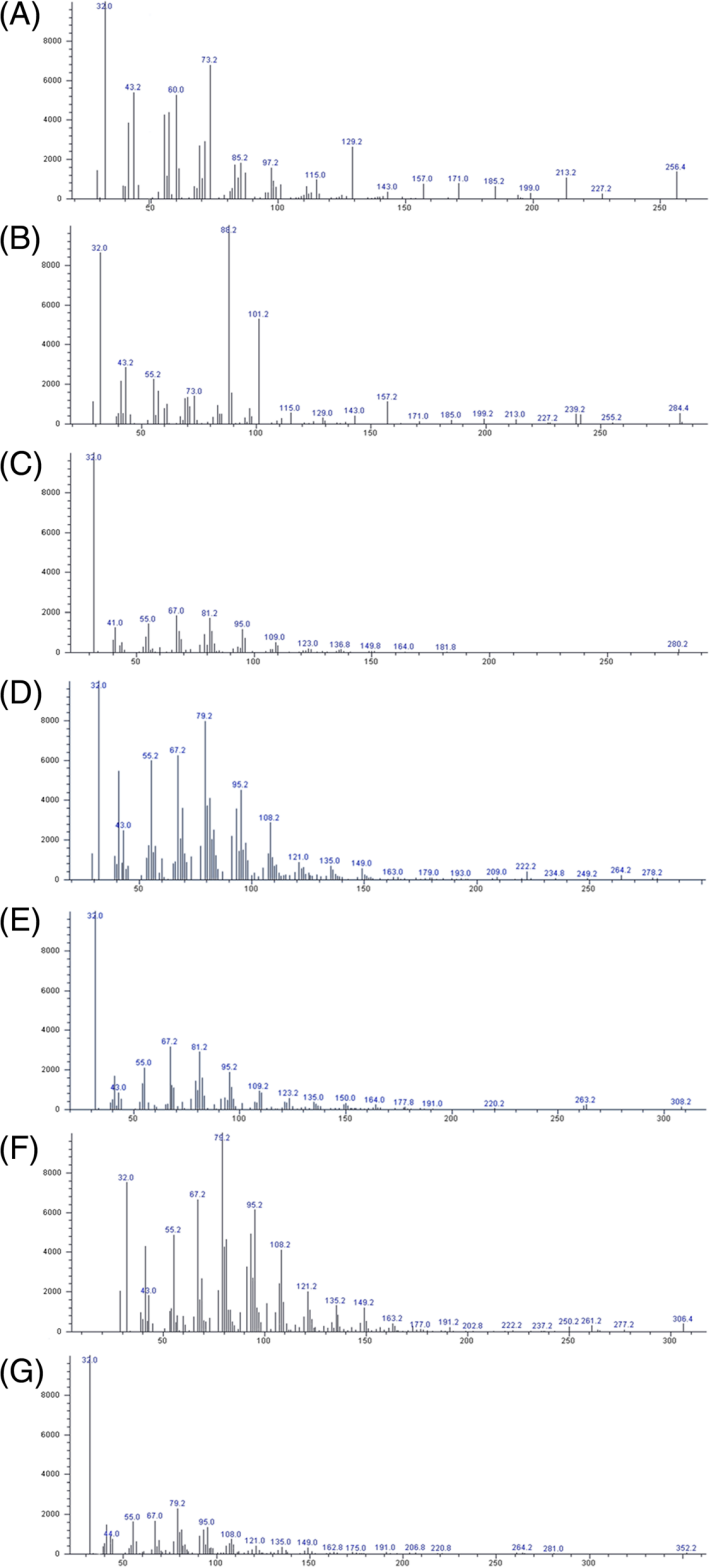
**MS Spectrum of the ethanol extract of *Prunella vulgaris *var. *lilacina*.** The X-axis and Y-axis of the chromatogram show mass/charge (m/z) and abundance, respectively. **(A)** Hexadecanoic acid; **(B)** ethyl palmitate; **(C)** linoleic acid; **(D)** (z,z,z)-9,12,15-octadecatrienoic acid; **(E)** ethyl(9E,12E)-9,12-octadecadienoate; **(F)** (z,z,z)-ethyl ester-9,12,15-octadecatrienoic acid; **(G)** ethyl linoleolate.

In the present study, the results clearly demonstrate that the *P. vulgaris* var. *lilacina* ethanol extract induced significant cytotoxic effects on the various cancer cell lines studies, and these effects were stronger than for the *P. vulgaris* var. *lilacina* solvent fractions. It may be difficult to determine the contribution of individual components on the overall anticancer effects. In the literature, it has been reported that *P. vulgaris* var. *lilacina* components such as ursolic acid and rosmarinic acid are responsible for anticancer activities. Woo et al. [34] reported significant apoptogenic activity of 2α,3α-dihydroxyurs-12-ene-28-oic acid in Jurkat T cells. Lee et al. [35] and Hsu et al. [36] explored the cytotoxic effects of ursolic acid. Psotova et al. [37] found that rosmarinic acid from *P. vulgaris* var. *lilacina* exhibited strong anticancer activity. In the present study, we have isolated various presumed active compounds from the ethanol extract of *P. vulgaris* var. *lilacina* (Figures 4 and 5). The spectrum profile of GC-MS confirmed the presence of 7 major components, which were hexadecanoic acid, ethyl palmitate, lioleic acid, (z,z,z)-9,12,15-octadecatrienoic acid, ethyl (9E, 12E)-9,12-octadecadienoate, (z,z,z)-ethyl ester-9,12,15-octadecatrienoic acid, and ethyl linoleolate. Lai et al. [38] reported significant antitumor effects of fatty acids such as hexadecanoic acid and ethyl palmitate obtained from plant extracts. Taken together, the anticancer activity of ethanol extract may be the result of the synergistic effects of various compounds in *P. vulgaris* var. *lilacina*, which suggests that *P. vulgaris* var. *lilacina* can be used as a biological agent in the treatment of cancer.

### Real-time RT-PCR analysis

**Figure 6:**
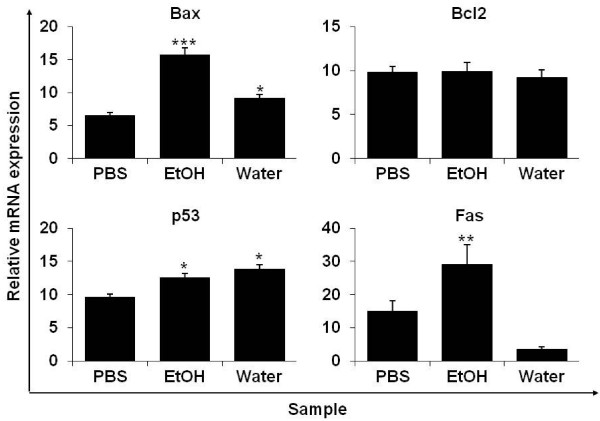
**mRNA expression of apoptotic genes in A549 cells treated with ethanol extracts and water fractions from *Prunella vulgaris *var. *lilacina*.** Cells were treated with the ethanol extract and water fraction of *P. vulgaris* var. *lilacina* at 10 μg/ml. Values are the mean ± SEM; different marks within treatments indicate significant differences (*p < 0.05, **p < 0.01, ***p < 0.001).

We assessed whether *P. vulgaris* var. *lilacina* ethanol extract and the water fraction affected the expression of genes associated with apoptotic cell death, including the tumor suppressor p53,pro-apoptotic Bax, the anti-apoptotic Bcl-2 and Fas genes in A549 cells. p53-mediated apoptosis primarily occurs through the intrinsic apoptotic program [39]. It was reported that p53 induces apoptosis by either increasing transcriptional activity of proapoptotic genes such as Bax or suppressing the activity of the antiapoptotic genes of the Bcl-2 family [40]. Our data show that *P. vulgaris* var. *lilacina* ethanol extract and the water fraction significantly increased the expression of p53, Bax and Fas compared to the control. However, the expression of Bcl-2 was not decreased compared to that of the control (Figure 6). Therefore, the treatments altered the expression of Bax/Bcl-2, resulting in a shift in their ratio favoring apoptosis. Several other groups have shown in various cancer cell lines that *P. vulgaris* var. *lilacina* can lead to cell death by inducing apoptosis through regulation of p53 and Bax/Bcl-2 expression [41]. In our study, the resulting elevation in p53 and Bax protein expression in lung cancer cells is consistent with our earlier proposed involvement of p53 and Bax-related response systems. Taken together, we suggest that *P. vulgaris* var. *lilacina* ethanol extract and water fraction induce apoptosis through the regulation of p53, Bax, and Fas expression.

## Conclusions

The present study determined that *P. vulgaris* var. *lilacina* extract and its fractions have strong antioxidant and anticancer activities *in vitro*. The correlation coefficients between antioxidant capacity and the phenolic content were very strong, and phenolic compounds were a major contributor to the antioxidant capacities of *P. vulgaris* var. *lilacina*.

In addition, we confirmed the presence of 7 major components of *P. vulgaris* var. *lilacina*. However, further studies to study the mechanisms of these compounds and find the root of their antioxidative and anticancer activity are underway. On the basis of these results, *P. vulgaris* var. *lilacina* appears to be a good source of natural antioxidant and anticancer agents and could be of significance in the food industry and for the control of various human and animal diseases.

## Abbreviations

ABTS: 2, 2′-Azinibis 3-ethyl benzothiazoline-6-sulfonic acid; DCF-DA: Dichlorofluorescein diacetate; DMSO: Dimethyl sulfoxide; DPPH: 1, 1-diphenyl-1-picrylhydrazyl; FRAP: Ferric-reducing antioxidant power; GAE: Gallic acid equivalent; LPS: Lipopolysaccharide; ROS: Reactive oxygen species; SOD: Superoxide dismutase; TPTZ: 2, 4, 6-tris(2-pyridyl)-s-triazine.

## Competing interests

The authors declare that they have no competing interests.

## Authors’ contributions

KAH conceived this study and designed the experiments. YJH and EJL performed most of the experiments. All authors including HRK analyzed the data and discussed the results. KAH supervised the project and wrote the manuscript with the help of YJH, EJL and HRK, and all authors read and approved the final manuscript.

## Pre-publication history

The pre-publication history for this paper can be accessed here:

http://www.biomedcentral.com/1472-6882/13/310/prepub

## Supplementary Material

Additional file 1: Table S1 Extraction yield of *Prunella vulgaris* var. *lilacina*. Click here for file
