# Supplementary figures and images for: In vitro antioxidant and anticancer effects of solvent fractions from prunella vulgaris var. lilacina
Source: BMC Complement Altern Med. 2013 Nov 9;13:310. doi: 10.1186/1472-6882-13-310 (PMC4226201; doi:10.1186/1472-6882-13-310)

**Supplementary table 1. Extraction yield of *Prunella vulgaris var. lilacina.***


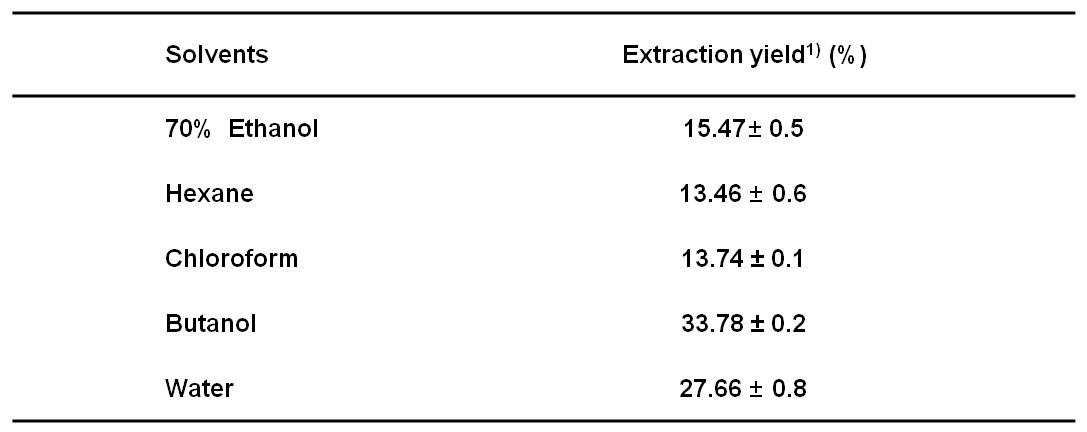


**1) Values are mean ± SEM.**

Supplement: Additional file 1: Table S1 — Extraction yield of Prunella vulgaris var. lilacina. [file 1472-6882-13-310-S1.docx]
